# Predicting Long-Term Prognosis of Poststroke Dysphagia with Machine Learning

**DOI:** 10.3390/jcm14145025

**Published:** 2025-07-16

**Authors:** Minsu Seo, Changyeol Lee, Kihwan Nam, Bum Sun Kwon, Bo Hae Kim, Jin-Woo Park

**Affiliations:** 1 Department of Physical Medicine & Rehabilitation, Dongguk University College of Medicine, Goyang 10326, Republic of Korea; seoms87@dumc.or.kr (M.S.); bskwon@dumc.or.kr (B.S.K.); 2 Department of Data Analysis, AimedAI, Seoul 06178, Republic of Korea; charlie@aimed.ai; 3 Graduate School of Management of Technology, Korea University, Seoul 06178, Republic of Korea; namkh@korea.ac.kr; 4 Department of Otorhinolaryngology-Head and Neck Surgery, Dongguk University Ilsan Hospital, Goyang 10326, Republic of Korea; bohae111@naver.com

**Keywords:** deglutition, machine learning, stoke, prognosis

## Abstract

**Background**: Poststroke dysphagia is a common condition that can lead to complications such as aspiration pneumonia and malnutrition, significantly affecting the quality of life. Most patients recover their swallowing function spontaneously, but in others difficulties persist beyond six months. Can we predict this in advance? On the other hand, there have been recent attempts to use machine learning to predict disease prognosis. Therefore, this study aims to investigate whether machine learning can predict the long-term prognosis for poststroke dysphagia using early videofluoroscopic swallowing study (VFSS) data. **Methods**: Data from VFSSs performed within 1 month of onset and swallowing status at 6 months were collected retrospectively in patients with dysphagia who experienced their first acute stroke at a university hospital. We selected 14 factors (lip closure, bolus formation, mastication, apraxia, tongue-to-palate contact, premature bolus loss, oral transit time, triggering of pharyngeal swallow, vallecular residue, laryngeal elevation, pyriform sinus residue, coating of the pharyngeal wall, pharyngeal transit time, and aspiration) from the VFSS data, scored them, and analyzed whether they could predict the long-term prognosis using five machine learning algorithms: Random forest, CatBoost classifier, K-neighbor classifier, Light gradient boosting machine, Extreme gradient boosting. These algorithms were combined through an ensemble method to create the final model. **Results**: In total, we collected data from 448 patients, of which 70% were used for training and 30% for testing. The final model was evaluated using accuracy, precision, recall, F1-score, and Area Under the Receiver Operating Characteristic Curve (AUC), resulting in values of 0.98, 0.94, 0.84, 0.88, and 0.99, respectively. **Conclusions**: Machine learning models using early VFSS data have shown high accuracy and predictive power in predicting the long-term prognosis of patients with poststroke dysphagia, and they are likely to provide useful information for clinicians.

## 1. Introduction

Dysphagia is a common complication in acute stroke, with a reported prevalence ranging from 32.1% to 69.6% [1,2]. Aspiration pneumonia, dehydration, and malnutrition are frequent complications of dysphagia, significantly impacting stroke patients’ mortality rates, length of hospital stay, and overall rehabilitation outcomes [3]. While most patients regain some swallowing ability within one month poststroke, 11% to 50% of patients continue to experience dysphagia beyond six months [4,5,6]. Therefore, predicting the prognosis of poststroke dysphagia is crucial for developing appropriate treatment plans. However, predicting dysphagia outcomes remains challenging due to the complex and multifactorial nature of the condition, and, currently, predictions rely heavily on clinical judgment and limited criteria [7].

Recently, there has been an increasing interest in utilizing machine learning to predict disease outcomes in the medical field. Machine learning has shown excellent performance in identifying patterns and generating predictive models from complex medical data [8]. In stroke research, machine learning has been successfully applied to predicting stroke occurrence [9], diagnosis and outcome [10,11], and poststroke activities of daily living [12]. Given these achievements in stroke-related research, machine learning has the potential to improve dysphagia prognosis predictions, overcoming the challenges of clinical complexity and variability.

Several previous studies have explored the prediction of poststroke dysphagia using clinical or imaging-based approaches. Galovic et al. [13] developed a model incorporating age, NIHSS score, and aspiration risk to estimate oral intake recovery. Dubin et al. [14] predicted the need for enteral feeding based on early clinical parameters such as age and lesion location. Additionally, Lee et al. [15] used a Bayesian network that included lesion laterality and initial severity of dysphagia to forecast recovery. More recently, Ye et al. [16] proposed a machine learning model using clinical features such as NIHSS score, Barthel Index, age, and lesion location to predict severe dysphagia in ischemic stroke patients. Their XGBoost-based model achieved a high predictive performance, highlighting the feasibility of data-driven risk stratification early after stroke. Furthermore, Park et al. [17] developed an aspiration screening tool using machine learning algorithms trained on large-scale hospital datasets. Their model, designed for real-time clinical application, outperformed conventional screening tools such as the Gugging Swallowing Screen. However, these models did not utilize detailed physiological assessments of swallowing such as videofluoroscopic swallowing studies (VFSSs). The VFSS is considered the gold standard for dysphagia assessment [18]. To quantify VFSS results and provide an objective measure of dysphagia severity, clinicians often use the videofluoroscopic dysphagia scale (VDS), a validated tool that evaluates various elements of the oral and pharyngeal phases [19,20]. Han et al. [21] have attempted to use VDS to predict the prognosis of dysphagia that persists beyond six months poststroke, reporting that VDS parameters significantly correlate with recovery in poststroke dysphagia patients.

This study aims to investigate whether a machine learning model based on the initial VFSS data collected within a month of the onset of the first stroke can predict the long-term prognosis of poststroke dysphagia patients at six months.

## 2. Materials and Methods

### 2.1. Study Design

We conducted a retrospective study of patients who experienced their first acute stroke and were diagnosed with dysphagia via VFSS from January 2014 to November 2023. The inclusion criteria for this study were as follows: (1) first-ever acute stroke (ischemic or hemorrhagic) confirmed by magnetic resonance imaging (MRI) or computed tomography (CT), (2) dysphagia diagnosed through VFSS, and (3) patients aged 20 years or older. We excluded patients with other organic brain diseases, such as neurodegenerative or neuromuscular disorders, that could independently impact swallowing function. The study was approved by the Institutional Review Board of Dongguk University Ilsan Hospital (No. 2023-10-004). All the procedures were conducted following the relevant guidelines and regulations.

### 2.2. Data Collection and Outcome Measures

Demographic data, neurological characteristics, and swallowing function were extracted from the electronic medical records, including age, sex, stroke type (ischemic or hemorrhagic), VFSS findings (conducted within one month of stroke onset), and swallowing status at six months poststroke. The VFSS and its interpretation were based on Logemann’s procedure [22] and were performed by two clinicians: one with over 20 years of experience and the other with 6 years of experience. Patients were given 5 mL of diluted barium (35% weight/volume) twice, and in cases of severe swallowing impairment, scores were assigned for evaluation. The final conclusions were drawn by consensus.

The swallowing function was assessed using the VDS, which evaluates 14 items divided into oral (7 items) and pharyngeal phases (7 items). These included lip closure, bolus formation, mastication, apraxia, tongue-to-palate contact, premature bolus loss, oral transit time, triggering of pharyngeal swallow, vallecular residue, laryngeal elevation, pyriform sinus residue, coating of the pharyngeal wall, pharyngeal transit time, and aspiration.

Each parameter was assigned an ordinal score based on standardized operational definitions, reflecting increasing degrees of dysfunction. For example, lip closure was scored as 0 (intact), 1 (inadequate), or 2 (none); premature bolus loss as 0 (none), 1 (<10%), 2 (10–50%), or 3 (>50%). Similar scoring definitions were applied for residue measures based on bolus percentage. These scoring criteria were derived from previously validated clinical protocols and established through expert consensus. The resulting ordinal variables served as structured input features for the machine learning models and are detailed in Table 1. The primary outcome measure was the swallowing function at six months poststroke, as documented in clinical follow-up records or through repeat VFSS, if clinically indicated. The dysphagia prognosis was evaluated using the Functional Oral Intake Scale (FOIS), a validated 7-point ordinal scale ranging from 1 (nothing by mouth) to 7 (total oral diet with no restrictions) [23]. We defined recovery as FOIS ≥ 4 (oral intake with minimal restrictions) and persistent dysphagia as FOIS ≤ 3 (tube-dependent with minimal amounts of food or liquid). Accordingly, recovery was scored as 0, and persistent dysphagia was scored as 1, to create binary outcome labels for model training. The dataset features are shown in Table 1.

### 2.3. Data Preprocessing

All the features in the dataset were categorical, so one-hot encoding was applied to convert these variables into numerical values for the machine learning models. One-hot encoding was chosen to represent the categorical data in a format that enhances model interpretability and performance. The parameters from the VDS, such as lip closure (intact, inadequate, none), were one-hot encoded and converted into separate binary features. The dysphagia prognosis was used as the label for the machine learning models, representing the outcome variable to be predicted. Specifically, the dysphagia prognosis was categorized into two classes: recovery (FOIS ≥ 4) and persistent dysphagia (FOIS ≤ 3). We encoded these outcomes as binary labels, with recovery assigned as 0 and persistent dysphagia as 1. This ensured that each category was treated independently in the machine learning model. We then normalized the features to maintain consistency across variables, preventing any single feature from disproportionately influencing the model due to differences in scale. Among the 448 patients, 396 were classified as recovered (FOIS ≥ 4) and 52 as having persistent dysphagia (FOIS ≤ 3). We randomly allocated the entire dataset into 70% training and 30% test sets using stratified random sampling to ensure the proportional representation of the outcome classes in both sets. A synthetic minority oversampling technique was applied to balance the target classes of the training dataset.

### 2.4. Machine Learning Models

After preprocessing, we used sixteen classification algorithms and chose five algorithms based on accuracy: Random forest (RF), CatBoost classifier (CBC), Light gradient boosting machine (LGBM), K-neighbor classifier (KNN), and Extreme gradient boosting (XGBoost). In order to optimize the model performance, we applied grid search for hyperparameter tuning. The search space for each algorithm was predefined based on the prior literature on medical AI [24,25,26,27], as well as on pilot testing on our dataset. For example, learning rates were selected within the commonly effective range of [0.01–0.15], and tree depths between 3 and 9 were evaluated to balance model complexity and overfitting risk. Each model underwent 50 iterations of grid search, and the parameter set that achieved the highest validation accuracy during internal cross-validation was selected. We applied 3-fold cross-validation on the training set for all five machine learning models. The detailed hyperparameters for each model are provided in Supplementary Material Table S1. Each model’s performance was compared to assess which algorithm offered the best accuracy for predicting the long-term prognosis of dysphagia. The performance of the models was evaluated using accuracy, area under the curve (AUC), precision, recall, and F1-score on the test set.

### 2.5. Ensemble Learning

After conducting the learning with each algorithm, we made a final algorithm by combining the algorithms into one through the ensemble method [28]. The ensemble method is a method of combining multiple algorithms into one and then deriving one result. We obtain the final result by majority voting across the outputs of the five algorithms. The advantages of the ensemble method include improved performance, reduced overfitting, and enhanced robustness . The ensemble method can achieve a high performance beyond the limitations of a single algorithm. With this method, we can expect consistent performance improvements, especially with various data and conditions. Also, this method can effectively prevent the issue of overfitting to particular data points. In addition, as the diversity of the algorithms increases, the stability of the prediction increases, which can lead to performance improvements.

### 2.6. Statistical Analysis

Statistical analyses were performed using Python 3.8 on Ubuntu 22.04 with the Scikit-learn (version 1.5) and SciPy (version 1.11.4) libraries. A receiver operating characteristic (ROC) curve analysis was conducted, and the AUC was calculated to evaluate the model performance. The confidence interval for the average AUC was calculated using bias-corrected and accelerated bootstrapping.

## 3. Results

### 3.1. Patient Characteristics

A total of 448 patients met the inclusion criteria for this study. The mean age of the patients was 69 ± 13 years, with 248 males (55.4%) and 200 females (44.6%). With regard to stroke types, 320 patients (71.4%) had ischemic strokes, and 128 patients (28.6%) had hemorrhagic strokes. The average duration from stroke onset to the first VFSS was 18.7 ± 17.5 days. Table 2 summarizes the baseline demographic and clinical characteristics.

### 3.2. Model Performance

**Figure 1:**
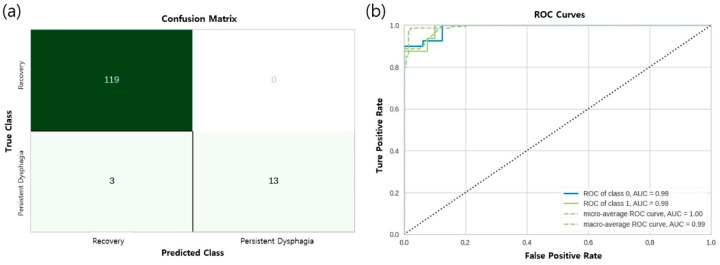
Confusion matrix and receiver operating characteristic (ROC) curve of the final ensemble model. (**a**) Confusion matrix of the final ensemble model. (**b**) Receiver operating characteristic (ROC) curve of the final ensemble model; area under the curve (AUC) = 0.99.

The machine learning models were evaluated using the test set, and their performance metrics are summarized in Table 3. Among the models, excluding the final ensemble model, the CBC achieved the highest accuracy (96%), followed by the XGBoost (95%) and RF (94%). The LGBM had the lowest accuracy among the top models at 93%, while KNN also achieved 94% accuracy but with a lower AUC value. The KNN showed limitations in handling the complex feature space, which contributed to its relatively lower AUC value. The AUC values for each model ranged from 0.88 to 0.99, indicating strong model discrimination ability. The final ensemble model, which combined the results of the five algorithms, achieved an accuracy of 98%, an AUC of 0.99, a precision of 0.94%, a recall of 0.84%, and an F1-score of 0.88, demonstrating the best overall performance. Figure 1 provides the confusion matrices and ROC curves for the final model. The figure allows for a detailed comparison of the classification performance and the discriminative ability.

## 4. Discussion

This study demonstrates that machine learning models using early VFSS data can achieve high accuracy and predictive power in predicting the long-term prognosis of poststroke dysphagia. The final ensemble model demonstrated a high predictive performance with an accuracy of 0.98, an AUC of 0.99, a precision 0.94, a recall of 0.84, and an F1-score of 0.88. The model’s accuracy of 98% shows that it correctly predicts the long-term swallowing outcomes for the majority of patients. This makes it a reliable tool in clinical settings, as it consistently provides accurate predictions. The AUC of 0.99 suggests that the model is highly effective at distinguishing between patients who will recover from dysphagia and those who will continue to experience difficulties. This means the model has excellent discriminative ability. The precision of 0.94 indicates that, when the model predicts recovery, it is correct 94% of the time. This reduces the likelihood of incorrectly identifying patients as recovering when they are not. However, the recall of 0.84 means the model may miss some patients who will continue to have swallowing difficulties, as it correctly identifies 84% of those with persistent dysphagia. The balance between precision and recall is reflected in the F1-score of 0.88, showing the model performs well overall in predicting outcomes accurately.

In previous studies, prognostic models have been developed to predict poststroke dysphagia outcomes. Dubin et al. [14] established a model to predict the need for enteral feeding using variables such as age, NIHSS score, and lesion location within the first 24 h of hospitalization, reporting an overall accuracy of 0.79. Galovic et al. [13] developed a prediction model for oral intake recovery, incorporating five factors including age, NIHSS score, and aspiration risk, and achieved an AUC of 0.82. Additionally, WH Lee et al. [15] used a Bayesian network to predict swallowing recovery after ischemic stroke, identifying initial dysphagia severity and bilateral lesions in specific brain regions as key predictors, with a reported AUC of 0.85. More recently, Ye et al. developed a machine learning model based on clinical features such as NIHSS score, Barthel Index, age, and lesion location to predict severe dysphagia in ischemic stroke patients [16]. Similarly, Park et al. trained a machine learning model using large-scale hospital data to predict aspiration in acute stroke patients. Their model outperformed conventional screening tools such as the Gugging Swallowing Screen, achieving an AUC of 0.81 [17]. However, both models primarily relied on general clinical features or structured screening tools and did not incorporate physiologic assessments such as the VFSS.

In contrast to previous studies that primarily relied on clinical characteristics (e.g., NIHSS scores, age, lesion location) or structural imaging data to predict swallowing outcomes, our study employed detailed ordinal parameters derived from VFSSs, using the validated VDS. By incorporating these physiologically grounded features into machine learning models, we were able to capture subtle patterns in the swallowing function that are not easily accessible through clinical variables alone. This approach allowed us to build a data-driven, physiology-based predictive model using the VFSS data obtained within one month poststroke. As such, our model provides a novel tool to support clinicians in early prognosis estimation and in the formulation of individualized treatment strategies for dysphagia management.

A previous study has identified key videofluoroscopic prognostic factors influencing poststroke swallowing function recovery and developed long-term prognostic tools based on these findings. Through logistic regression analysis, they examined the relationship between the initial VFSS data and the dysphagia prognosis at six months, developing the VDS based on the odds ratios of the prognostic factors [21]. Our research extends on this analysis by integrating these VDS parameters into machine learning models, learning complex patterns to achieve superior predictive performance.

The model’s strong performance makes it highly useful for managing poststroke dysphagia in clinical settings. Its ability to accurately distinguish between recovery and persistent dysphagia allows clinicians to prioritize high-risk patients for early, intensive rehabilitation, reducing complications like aspiration pneumonia and improving outcomes. The model also supports personalized treatment plans, where lower-risk patients can follow standard rehabilitation, and higher-risk patients receive more focused care. It can also assist with discharge planning, helping determine when patients are ready for home care or when they need extended inpatient or outpatient support, minimizing the risk of readmission. Additionally, by providing clear recovery expectations, the model helps clinicians set realistic goals and engage patients more actively in their care, improving adherence and satisfaction. Overall, this machine learning model can improve care, offering more-personalized and timely interventions for poststroke dysphagia patients.

This study has some limitations. First, this study was conducted using data from a single institution, which may limit the generalizability of the results to other populations or clinical settings. Second, the study did not control for the effects of swallowing therapy. Some patients received swallowing therapy while others did not, and this variable was not accounted for, which may have influenced the model’s accuracy. Future models should incorporate detailed information about the rehabilitation interventions to more accurately predict the dysphagia outcomes. Third, while the VDS provides a comprehensive assessment of swallowing function based on the VFSSs, other critical clinical factors, such as neurological damage severity, general health conditions, and cognitive function, were not included. These factors can significantly affect dysphagia recovery, and their exclusion may limit the model’s predictive capability. Expanding the feature set to include broader clinical and neurological data could improve both the model’s accuracy and its clinical relevance. Fourth, the variability in the VFSS timing is a notable limitation, as it was not standardized due to the retrospective design. Since the VFSS timing may reflect different stages of recovery, this could introduce confounding effects. Future prospective studies with a standardized VFSS timing are warranted to address this issue. Fifth, the use of a dichotomous outcome classification—defining recovery as FOIS ≥ 4 and persistent dysphagia as FOIS ≤ 3—may reduce the model’s sensitivity to intermediate swallowing outcomes, particularly in patients with partial recovery who remain on modified diets. Future research should consider adopting multi-class classification frameworks incorporating the full FOIS spectrum to enable a more nuanced prognosis and to facilitate individualized clinical decision-making. Furthermore, the cross-validation strategy used in this study involved only 3-folds, which may limit the robustness of the model evaluation. This choice was made to maintain a balance between the need for model validation and the retention of sufficient data in each fold, given the relatively small sample size (*n* = 448). Future studies with larger datasets should consider employing a greater number of folds, such as 5-fold or 10-fold cross-validation, to enhance the robustness and stability of model performance assessments. Finally, while the ensemble method improved model performance, there is a potential risk of overfitting due to the complexity of the algorithms used. Overfitting may cause the model to perform well on the training data but less effectively on new, unseen data. To mitigate this, more-robust techniques, such as cross-validation, should be employed in future research.

## 5. Conclusions

This study demonstrates the potential of machine learning models using early VFSS data to predict the long-term prognosis of poststroke dysphagia. The final ensemble model, with its high accuracy and predictive power, offers a promising tool for clinicians to identify patients at risk for prolonged dysphagia. This allows for timely interventions and personalized treatment plans, improving patient outcomes.

## Figures and Tables

**Table 1:** Dataset features.

Parameter	Operational Definition	Score
Lip closure	Complete lip seal during oral phase	0
	Incomplete seal; mild leakage observed	1
	No seal	2
Bolus formation	Well-formed cohesive bolus	0
	Partially formed; weak cohesion	1
	No cohesive formation	2
Mastication	Normal chewing pattern	0
	Incomplete or weak mastication	1
	No mastication	2
Apraxia	No signs of oral apraxia	0
	Mild impairment in voluntary oral movements	1
	Moderate impairment; inconsistent oral motor coordination	2
	Severe apraxia; inability to initiate or sequence oral actions	3
Tongue to palate contact	Full contact during bolus propulsion	0
	Weak or partial contact	1
	No contact; ineffective oral propulsion	2
Premature bolus loss	No bolus spillage into pharynx before swallow initiation	0
	<10% of bolus spills prematurely	1
	10–50% of bolus spills prematurely	2
	>50% of bolus spills prematurely	3
Oral transit time	Bolus transfer completed within 1.5 s	0
	Prolonged oral transit > 1.5 s	1
Pharyngeal delay time	Initiated within 0.5 s after bolus reaches ramus of mandible	0
	Delayed beyond 0.5 s	1
Vallecular residue	No residue	0
	<10% of bolus remains	1
	10–50% remains	2
	>50% remains	3
Laryngeal elevation	Normal elevation during swallowing	0
	Reduced elevation	1
Pyriform sinus residue	No residue	0
	<10% of bolus remains	1
	10–50% remains	2
	>50% remains	3
Coating of pharyngeal wall	No coating observed post-swallow	0
	Coating present	1
Pharyngeal transit time	<1.0 s	0
	>1.0 s	1
Aspiration	No penetration or aspiration	0
	Penetration above vocal folds without aspiration	1
	Aspiration below vocal folds, with or without cough reflex	2
Outcome	Recovery	0
	Persistent dysphagia	1

**Table 2:** Patient characteristics.

Characteristic	*n* = 448
Age	69 ± 13
Gender (M/F)	248/200
Etiology (*n*)	
Infarction	320
Hemorrhage	128
Days from onset to 1st study (days)	18.7 ± 17.5

Values are presented as mean ± standard deviation.

**Table 3:** Performance of prediction models.

	Accuracy	Precision	Recall	F1-Score	AUC
Random forest	0.94	0.89	0.80	0.84	0.96
CatBoost classifier	0.96	0.94	0.84	0.88	0.98
Light gradient boosting	0.93	0.86	0.80	0.83	0.96
K-neighbor classifier	0.94	0.89	0.80	0.84	0.88
Extreme gradient boosting	0.95	0.90	0.84	0.86	0.95
Final ensemble model	0.98	0.94	0.84	0.88	0.99

## Data Availability

The data underlying in this article will be shared upon a request to the corresponding author.

## Supplementary Materials

The following supporting information can be downloaded at https://www.mdpi.com/article/10.3390/jcm14145025/s1: Table S1: Tuned hyperparameters and searching methods for each machine learning model. Table S2: Summary of Model Performance (Mean ± SD).
