# Supplementary material for: Predicting Long-Term Prognosis of Poststroke Dysphagia with Machine Learning
Source: J Clin Med. 2025 Jul 16;14(14):5025. doi: 10.3390/jcm14145025 (PMC12295981; doi:10.3390/jcm14145025)
Supplement: Supplementary file 1 [file jcm-14-05025-s001.zip › Supplementary Material Table S1.pdf]

Supplemental Material

Supplementary Material Table S1. Tuned hyperparameters and searching methods for each machine learning model.

	Searching methods	Search space	Optimal hyperparameters for each model
Random forest	Grid	max_depth: [3, 5, 7, 9]	Iteration = 50; max_depth=7
CatBoost classifier	Grid	iterations: [10, 30, 50, 70]	Iteration = 50
Light gradient boosting	Grid	learning_rate: [0.01, 0.05, 0.1, 0.15]; number_of_leaves: [6, 12, 24] alpha: [1e-09, 1e-07, 1e-05]; lambda: [1, 10, 100]	Iteration = 50; learning_rate = 0.1; alpha = 1e-07; lambda = 10; number of leaves = 6
K-neighbors classifier	Grid	n_neighbors: [5, 10, 20, 28, 30]	Iteration = 50; n_neighbors = 28
Extreme gradient boosting	Grid	learning_rate: [0.01, 0.05, 0.1, 0.15]; max_depth: [3, 5, 7]	Iteration = 50; learning_rate = 0.15; max_depth = 5
